# Dystrophin-gene mutation location influences severity of electroretinogram defects in mouse models of Duchenne muscular dystrophy

**DOI:** 10.1186/s12916-026-04873-1

**Published:** 2026-04-25

**Authors:** André Maurício Passos Liber, Mirella Barboni, Yoshitsugu Aoki, Jan Kremers, Cyrille Vaillend

**Affiliations:** 1 Université Paris-Saclay, CNRS, Institut des Neurosciences Paris-Saclay, 91400 Saclay, France; 2 Department of Ophthalmology, Semmelweis University, Budapest, Hungary; 3 Institute of Molecular and Clinical Ophthalmology Basel (IOB), Basel, Switzerland; 4 Department of Molecular Therapy, National Institute of Neuroscience, National Center of Neurology and Psychiatry, Kodaira, Tokyo 187-8502 Japan; 5 Section for Retinal Physiology, University Hospital Erlangen, Erlangen, Germany

**Keywords:** Retina, Dystrophins, Mouse models, Duchenne muscular dystrophy, Electroretinogram, Genotype–phenotype relationships

## Abstract

**Background:**

Duchenne muscular dystrophy (DMD) results from mutations in the *DMD* gene, which differentially affect dystrophin isoforms (Dp427, Dp260, Dp140, Dp71) expressed in distinct brain and retinal cell types. The selective loss of one or more isoforms contributes to heterogeneous cognitive and neuropsychiatric comorbidities. Here, we investigated whether specific mutations differentially affect retinal function by comparing genotype-dependent electroretinographic (ERG) responses in mouse models lacking different dystrophins.

**Methods:**

We analyzed in vivo dark-adapted (DA) and light-adapted (LA) flash electroretinograms (ERG) in four adult DMD mouse models: *Mdx* and *mdx5cv* mice lacking Dp427; *mdx*^*2Cv*^ mice lacking Dp427 and Dp260; and *dmd-null* mouse lacking all dystrophins, compared to their respective WT littermate male mice and to ERGs previously recorded in *mdx52* mice (lacking Dp427, Dp260 and Dp140).

**Results:**

Mutations affecting the expression of the Dp140 and Dp71 isoforms produced more severe ERG abnormalities, consistent with findings in patients and aligned with intellectual disability severity. ERG parameter analysis revealed unique roles for Dp427 and Dp260 in rod ribbon-synapse transmission, additional Dp260 function in inner retina, and involvement of Dp140/Dp71 in cone photoreceptor pathways.

**Conclusions:**

These findings highlight the relevance of ERG as a potential biomarker for central dysfunction in DMD, and support its translational application for patient stratification and targeted therapeutic approaches.

**Supplementary Information:**

The online version contains supplementary material available at 10.1186/s12916-026-04873-1.

## Background

**Fig. 1:**
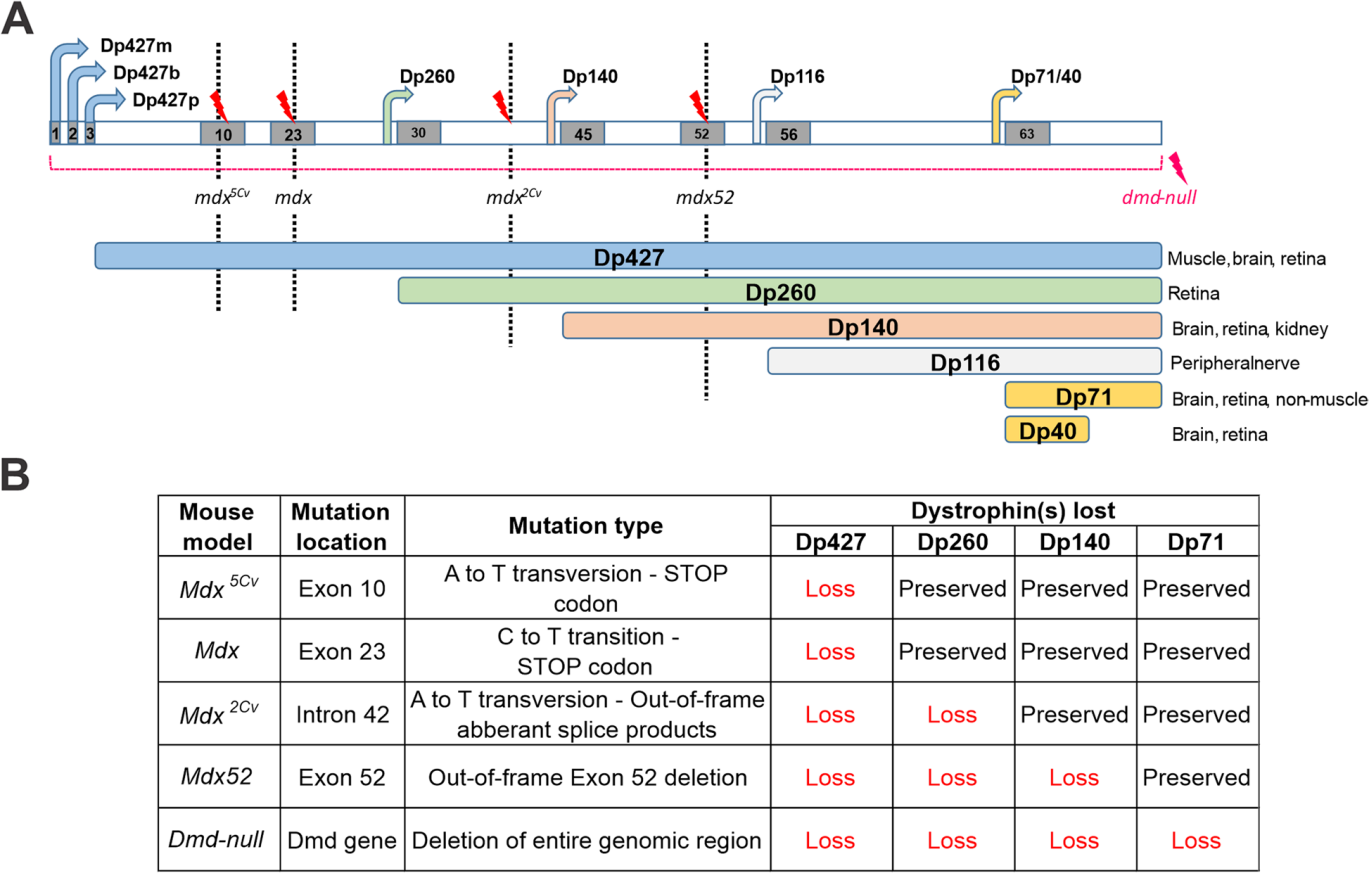
**(A)** Schematic representation of the *DMD* gene (Top), with exon numbers in grey boxes, promoter and transcription starts of each dystrophin isoform as angled arrows. Three specific promoters are shown for the full-length dystrophin: Dp427m (muscle form), Dp427b (brain form), and Dp427p (Purkinje cells). The corresponding dystrophin proteins of different sizes (Dp427, Dp260, Dp140, Dp116, Dp71, Dp40) are shown below (colors correspond with the color of promoter arrows). The main tissues expressing each isoform are listed on the right. The vertical dotted lines and red flashes show the position of the genetic variant of each mouse model, as indicated, except for dmd-null which have a complete deletion of the gene. **B** Table detailing the mutation location, mutation type and differential loss of isoforms for each mouse models

Duchenne muscular dystrophy (DMD; MIM: 310,200) is associated with severe muscular impairment and decreased life expectancy. DMD is caused by a mutation in the X-chromosomal *DMD* gene (MIM: 300,377) leading to absence of the full-length dystrophin protein Dp427. Depending on the location of the mutation, possibly other dystrophin isoforms, Dp260, Dp140 and Dp71 (the number indicating the molecular weight of the protein) are affected. These distinct dystrophins are produced by independent internal promoters (Fig. 1A). The further downstream the mutation is located on the *DMD* gene, the more dystrophins are lost. Mutations that prevent the expression of full-length and truncated forms of Dp427 are responsible for the muscular dystrophy. However, all dystrophins display specific patterns of expression in distinct cell types of the central nervous system (CNS), including in the retina (Table 1) [1–8], which is likely responsible for the diversity of the brain-related and retinal phenotypes in DMD patients [1, 9]. There is a positive relationship between severity of the cognitive impairment and number of lost dystrophins in patients [10] and a similar relationship has been proposed for retinal electrophysiology [11, 12].

**Table 1:** Known distribution of dystrophins mRNA and proteins in the retina

Retinal cell/layer	Dystrophins				
	**Dp427**	**Dp260**	**Dp140**	**Dp71**	**Dp40**
ONL	R + [8]	R + [8]	R + [8]	P + [19, 52]	
OPL	P + [8, 13, 14]	P + [8, 13, 14]		P + [19, 52]	
OPL (synaptic ribbon)	P + [8, 13, 14]	P + [8, 13, 14]			
Photoreceptors (R + C)	R + [5, 8]	R + [5] P + [8]			
Cone photoreceptors	R + [5, 8]	R + [5]	R + [5]		R + [5]
INL	R + [8]	R + [8]	R + [8]	P + [19, 52]	
INL (bipolar/amacrine cells)	R + [8]				
IPL				P + [19, 52]	
GCL	R + [25]			P + [19, 52]	
Müller glial cells and astrocytes				P + [17–19, 52]	

*R+* mRNA detected, *P+* Protein detected, *ONL* Outer nuclear layer, *OPL* Outer plexiform layer, *R+C* Rods and cones, *INL* Inner nuclear layer, *IPL* Inner plexiform layer, *GCL* Ganglion cell layer. This information is reviewed in [1]. Detailed identifications mentioned in the table can be found in the following publications as indicated: [5], [8], [13], [14], [17], [18], [19], [25], [52]

In mice, all dystrophins expressed in the brain are also expressed in the retina, which additionally expresses the retina-specific Dp260 isoform. The localization of each isoform in distinct retinal layers and cell-types, identified at the mRNA and/or protein levels, has been recently reviewed [1] and is detailed in Table 1. The mRNAs of Dp427, Dp260 and Dp140 were all detected in the outer nuclear layer (ONL) and inner nuclear layer (INL), suggesting putative expression in both photoreceptors and bipolar cells, but not in horizontal cells [8]. The Dp427 mRNA was more specifically localized in bipolar cells an some amacrine cells, and the protein was detected in the OPL with a possible enrichment in cones [8]. Dp260 is more strongly expressed than Dp427 in retina; its mRNA was detected in OPL [8], in both rod and cone purified photoreceptors [5], and the Dp260 protein has been localized at the photoreceptor ribbon synapse [13, 14]. The absence of Dp260 is claimed to be the cause of the ERG alterations in DMD [15, 16]. In contrast, little is known about the pattern of expression and function of Dp140 in the retina. However, its mRNA has been detected in cone-only flow-sorted photoreceptor samples from adult mouse retina [5]. Finally, the smallest *DMD* gene product, Dp71, displays a unique pattern of expression in the retina. The Dp71 protein can be detected across retinal layers, as it is expressed in Müller glial cells (MGC), retinal astrocytes and pericytes [17–19]. Its functions in the retina has been extensively investigated following the development of the Dp71-null mouse model with a selective loss of Dp71 [20–23]. However, genetic alteration exclusively affecting Dp71 is ultra-rare and the Dp71-null mouse specifically affecting Dp71 with preservation of other dystrophins do not cause DMD. Finally, Dp40 is expressed from the same promoter as Dp71 but its transcription ends at exon 70 due to an alternative polyadenylation signal; its mRNA has been detected in cone photoreceptors [5].

Mouse models and patients with genetic alterations of the *DMD* gene, presumably affecting the expression of one or multiple dystrophins, display functional deficits in the retina [1]. Flash ERGs from dystrophic mouse models, recorded by our group [16, 20, 24] and by others [13, 25–27], showed slight to severe changes depending on the mutation in the model. The cumulative loss of dystrophin proteins in the mouse retina was associated with more severe dark-adapted (DA) ERG alterations, with the most severe phenotype in human patients resembling the phenotype of *mdx*^*3Cv*^ mice lacking all dystrophins [13]. More recently, we revisited the *mdx*^*3Cv*^ ERG phenotype using a more complete and refined ERG protocol, including DA and light-adapted (LA) flashes as well as sinewave and sawtooth modulations. The results showed that ERGs, including the oscillatory potentials (OPs), were reduced in this model and, moreover, an asymmetric On and Off ERG dysfunction was observed [24]. However, in this model the expression of all dystrophins is diminished but not completely abolished. The selective loss of Dp427 in *mdx* or *mdx*^*5Cv*^ mice results in normal to minor ERG changes in young adult mice (12–16 weeks old), while more deteriorated ERG amplitudes are observed at 15–18 months of age [13, 25, 26]. The loss of Dp427 and Dp260 in *mdx*^*2Cv*^ mice, and that of Dp427, Dp260 and Dp140 in *mdx*^*4cv*^, was only studied in dark-adapted conditions, revealing delayed b-wave and OPs in both models [13, 14]. More recently, we reappraised the ERG profile of the *mdx52* model (lacking Dp427, Dp260 and Dp140, as in *mdx*^*4Cv*^ mice) using an extended repertoire of DA and LA stimuli, showing additional decreases in the amplitudes of b-wave, OPs, sawtooth and sinewave responses in various stimulation conditions [16]. The results suggest that, as with cognitive impairments associated with DMD, retinal physiology is affected differently depending on the number of dystrophins lost. Therefore, ERGs may represent a signature of central comorbidities, and exploring retinal dysfunction through non-invasive ERG recordings may prove useful for both the diagnosis and therapeutic monitoring of CNS-related dysfunctions in DMD. However, it is currently still challenging to draw definite conclusions regarding the relationship between the position of the mutation, the missing dystrophins and the severity of retinal defects, due to disparate studies using different mouse models, equipment and stimulation protocols.

The aim of the present study therefore was to provide a detailed genotype–phenotype correlation of retinal dysfunction in DMD and to specify the involvement of each retinal dystrophin in retinal pathology. We thus undertook an updated comparison of ERG defects in mouse models of DMD holding distinct mutation profiles (Fig. 1B), using the identical ERG recording conditions as applied in our previous study of *mdx52* mice [16] and Dp71-null mice [20]. We recorded DA and LA ERGs to a series of flash strengths from DMD mouse models lacking Dp427 (*mdx* and *mdx*^*5Cv*^), Dp427 and Dp260 (*mdx*^*2Cv*^), or all dystrophins (*dmd-null*). Detailed retinal phenotypes of these mouse models have not been described, nor have they been directly compared before. Here, we analyzed ERG changes in each mouse model relative to their respective wild-type littermate to provide an accurate comparison among DMD models, including the previously studied *mdx52* mouse [16]. This comparison leads to a detailed genotype–phenotype analysis of retinal dysfunctions in DMD.

## Methods

### DMD mouse models

Four different DMD mouse models lacking different dystrophins were used in this study (Fig. 1B):

*Mdx*—The *mdx* mutation was identified in the C57BL/10ScSn mouse line, as a C to T transition resulting in a termination codon within exon 23 and selective loss of Dp427 [28]. The Dmd^*mdx*^ allele was introgressed to the C57BL/6 J inbred line by Dr. Dongsheng Duan (University of Missouri School of Medicine, USA). Breeders were purchased from the Jackson Laboratory (JAX stock #41,196; Bar Harbor, ME, USA). The line was backcrossed with the C57BL/6 J parental line for 9 generations.

*Mdx*^*5Cv*^**—**This line generated by chemical mutagenesis [29] presents a single A to T transversion in exon 10 of the dystrophin genomic DNA, creating a new splice donor site leading to a frame shifting 53 bp deletion, a stop codon in the mRNA and selective loss of Dp427, similarly to the *mdx* mouse [30]. Breeders (B6Ros.Cg-*Dmd*^*mdx−5Cv*^/J) were purchased from the Jackson Laboratory (JAX stock #002379; Bar Harbor, ME, USA). This line was backcrossed with the C57BL/6 J parental line for over 15 generations.

*Mdx*^*2Cv*^—This line was generated by chemical mutagenesis [29]. The mutation is a single base change in the splice acceptor sequence of dystrophin intron 42, which leads to aberrant splicing events that do not preserve the normal open reading frame and prevent expression of Dp427 and Dp260 [30]. Breeders (B6Ros.Cg-Dmd^*mdx−2Cv*^) were purchased from the Jackson Laboratory (JAX stock #002388; Bar Harbor, ME, USA). The *mdx*^*2Cv*^ mouse line was backcrossed with the C57BL/6 J parental line for over 15 generations.

*Dmd-null*—This line lacking all dystrophins was produced by deletion of the entire genomic region of the DMD gene using a Cre-loxP recombination system [31]. The line was produced by Prof. Kazunori Hanaoka (Kitaso University), provided to author YA, then transferred and studied at CNRS (France). This mouse line was backcrossed for over 10 generations with the C57BL/6 J line.

To produce the different mouse models, heterozygous females were crossed with C57BL/6JRj male mice, resulting in progenies containing both mutant and littermate control males with a 50:50 ratio (wild-type, WT), which were used to form the experimental groups (Breeding scheme in Additional file 1: Fig. S1). The genotype was determined by PCR analysis of tail DNA.

Previous biochemical analyses have shown that the expression of Dp427 and Dp260 is lost in the photoreceptor-to-bipolar cell synapse (outer-plexiform layer) in *mdx*^*2Cv*^ and *mdx52* mice [13, 14], and all dystrophins are absent from both muscle and brain tissues in *dmd*-null mice [31]. We have confirmed the lack of immunoreactive signal for dystrophins in retinal sections of *dmd*-null mice. In a recent study [32], we also showed that there is no residual expression of the targeted dystrophins in *mdx5cv* and *mdx52* mice, i.e., no Dp427 expression in *mdx*^*5C*v^ mice, no expression of Dp427 and Dp140 in *mdx52* mice. Moreover, the Dp140 expression level was unchanged in *mdx*^*5C*v^ mice, and Dp71 levels were not affected in both *mdx*^*5C*v^ and *mdx52* models. Therefore, these models either express or lack one, several or all dystrophins, but do not show variable or residual levels in their expression.

ERG recordings were performed in young-adult mice at the age 3–4 months old. All mouse lines were kept under a standard 12 h light–dark cycle with food and water ad libitum.

### Experimental groups

ERGs were recorded from: 34 eyes of 17 *mdx* male mice (89.24 ± 6.28 days old) and 32 eyes of 16 WT male littermates (87.25 ± 4.96 days old), 34 eyes of 17 *mdx*^*5Cv*^ male mice (89.24 ± 6.28 days old) and 32 eyes of 16 WT male littermates (87.25 ± 4.96 days old), 32 eyes of 16 *mdx*^*2Cv*^ male mice (99.63 ± 3.16 days old) and 32 eyes of 16 WT male littermates (99.56 ± 3.14 days old), 28 eyes of 14 *dmd-null* male mice (118.21 ± 3.38 days old) and 24 eyes of 12 WT male littermates (117.83 ± 3.54 days old). Littermate WT controls were used to avoid bias due to putative environmental factors, cage effects and genetic polymorphisms that may remain even after several backcrosses to the C57BL/6 parental strain [33].

### Animal preparation

Protocols and procedures have been described previously [16, 20, 24]. Briefly, mice were dark-adapted for at least 12 h. Animal preparation and electrode placement were performed under deep red illumination to maintain the retina in a dark-adapted state. Anesthesia was performed by an intramuscular injection with a single dose of 10% ketamine (ketamine 1000; Virbac, France) and 2% xylazine (Rompun; Bayer Healthcare, Puteaux, France), 1:1—1 µl/g. During ERG recordings, animals were positioned on a water-heated platform (38 °C) to maintain body temperature during anesthesia. Subcutaneous injections of 0.9% saline (300 μl before recordings, 100 μl after recordings) were given to prevent dehydration. Pupils were fully dilated using eye drops of Tropicamide 2 mg (Mydriaticum; Thea, France) and 5% phenylephrine (Neo synephrine FAURE; Europhta, Monaco). Contact lens electrodes (Ø 3.2 mm; Mayo Corporation, Inazawa, Japan) filled with Ocry-gel (Dr. Mann Pharma, Berlin, Germany) were positioned on the corneas of the two eyes (active electrodes). To prevent cornea dehydration, after recordings, ocry-gel was placed once more on the cornea. Two needle electrodes (Concentric Subdermal Steel Needle; Roland Consult, Brandenburg, Germany) were placed subcutaneously medial to the two ears (reference electrodes) and one was positioned subcutaneously at the base of the tail (ground electrode).

### ERG recordings

Binocular recordings of full-field ERGs and stimulus presentations were performed with a RetiPort system (Roland Consult, Brandenburg, Germany) controlling a Ganzfeld stimulator (Q450SC, Roland Consult). Signals (with 250 ms of time recording) were amplified 100.000 times, band-pass filtered between 1 and 300 Hz, and digitized at a rate of 512 Hz. Sweeps containing artifacts were automatically excluded during recordings. ERGs were recorded in order of increasing mean luminance to the following stimulus conditions:

*Scotopic flashes* – Dark-adapted rod and mixed rod-cone mediated ERG responses were recorded to flashes (white light) of −3.7, −2.7, −1.7, −0.7, and 0.3 log cd.s/m^2^ strengths on a dark background with 12, 10, 8, 8, and 4 sweeps with 1, 2, 5, 10 and 20 s inter-stimulus time intervals respectively, while maintaining dark adaptation. Likewise, the interval between each condition increased (30, 30, 60 and 120 s) as the flash strength increased.

*Photopic flashes* – After 2 min of adaptation to a white background of 25 cd/m^2^, white flashes of 0.3 log cd.s/m^2^ strength were delivered upon the background. Twenty-four flashes were averaged with an interstimulus interval of 1 s.

### ERG signal processing and analysis

**Fig. 2:**
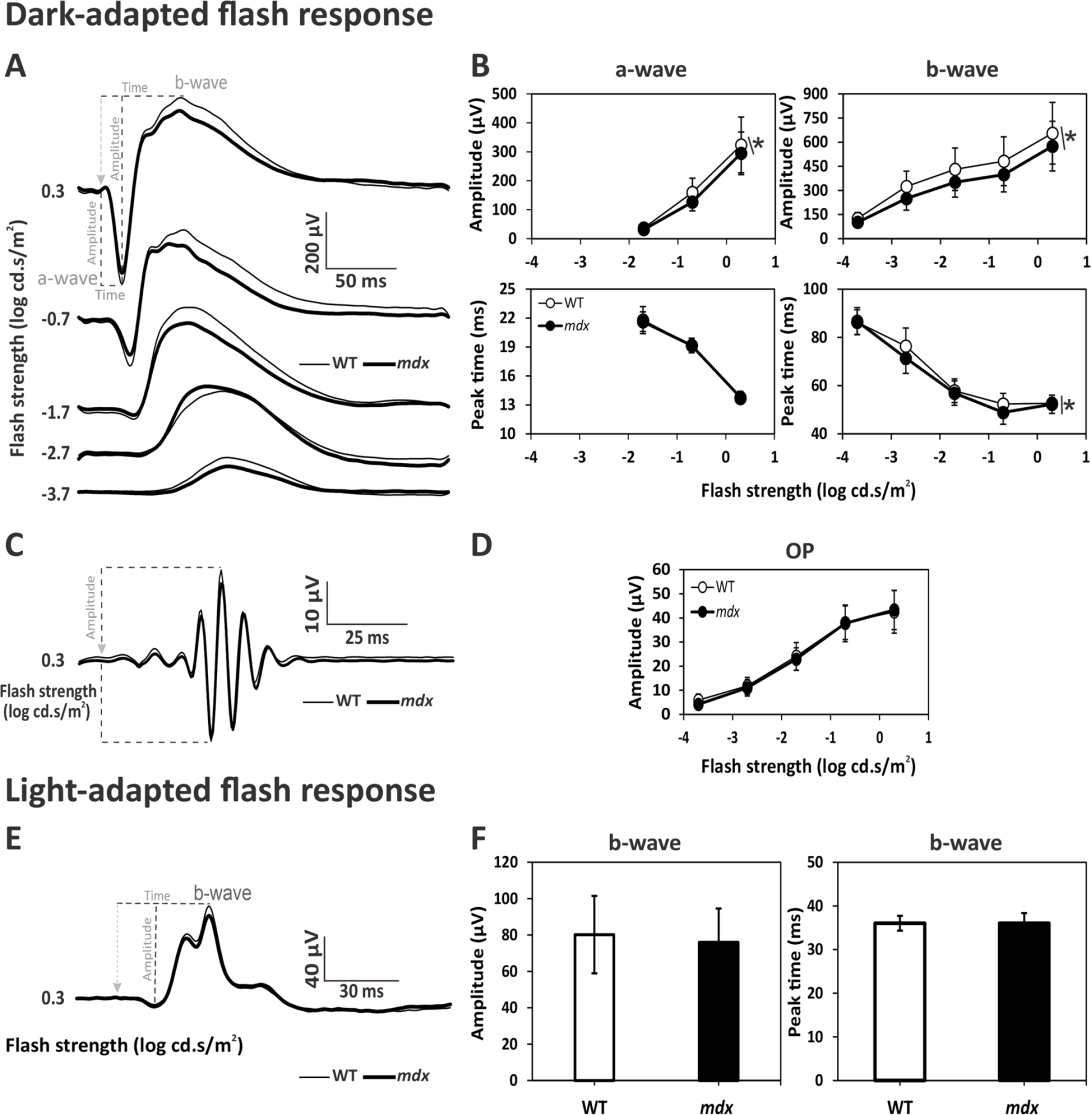
Dark-adapted (DA) and light-adapted (LA) flash ERGs in WT and *mdx* mice. **A** Averaged DA waveforms with OPs removed in WT (thin traces) and *mdx* mice (bold traces). Flash strength and definitions of key components (a-wave, b-wave) are indicated. **B** Mean (± SD) amplitudes (μV; upper plots) and implicit times (ms; lower plots) of DA a- and b-waves as a function of flash strength, in WT (open symbols) and *mdx* mice (filled symbols). **C** Averaged OP traces isolated from the strongest DA flash (0.3 log cd.s/m^2^) in WT (thin traces) and *mdx* mice (bold traces). **D** Mean (± SD) amplitude of OPs as a function of flash strength in WT (open symbols) and *mdx* (filled symbols) mice. **E** Averaged LA waveforms of WT (thin traces) and *mdx* mice (bold traces) at 0.3 log cd.s/m^2^ flash strength. **F** Histograms showing the mean (± SD) amplitude and implicit time of the LA b-wave. Dotted lines in A, C and E show the physiological hallmarks used for measurement of amplitudes and/or implicit times; the light-grey arrowhead marks the onset of the stimulus. Recordings were made in 32 eyes of 16 WT mice and 34 eyes of 17 *mdx* mice. Significant genotype effects (*p* < *0.05*) are marked with an asterisk

**Fig. 3:**
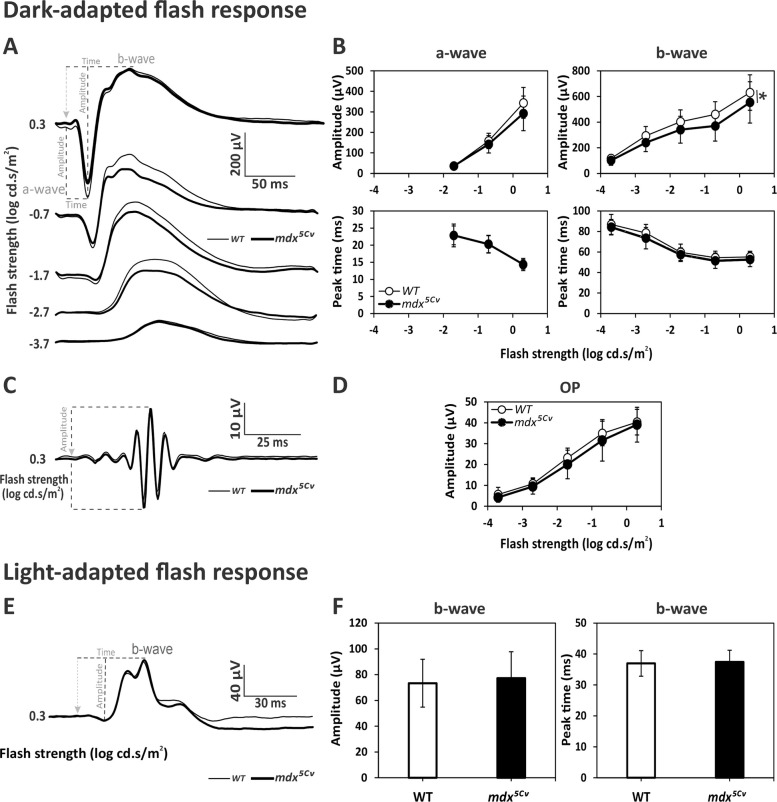
Dark-adapted (DA) and light-adapted (LA) flash ERGs of WT and *mdx*^*5Cv*^ mice. **A** Average DA waveforms with OPs removed in WT (thin traces) and *mdx*^*5Cv*^ (bold traces). Flash strength and definitions of key components (a-wave, b-wave) are indicated. **B** Mean (± SD) amplitudes (μV; upper plots) and implicit times (ms; lower plots) of DA a- and b-waves as a function of flash strength, in WT (open symbols) and *mdx*^*5Cv*^ mice (filled symbols). **C** Average OP traces isolated from the strongest DA flash (0.3 log cd.s/m^2^) in WT (thin traces) and *mdx*^*5Cv*^ (bold traces). **D** Mean (± SD) amplitude of OPs as a function of flash strength in WT (open symbols) and *mdx*^*5Cv*^ (filled symbols) mice. **E** Average LA waveforms of WT (thin traces) and *mdx*^*5Cv*^ (bold traces) at 0.3 log cd.s/m^2^ flash strength. **F** Histograms showing the mean (± SD) amplitude and implicit time of the LA b-wave. Dotted lines in A, C and E show the physiological hallmarks used for measurement of amplitudes and/or implicit times; the light-grey arrowhead marks the onset of the stimulus. Recordings made in 32 eyes of 16 WT mice and 34 eyes of 17 *mdx*^*5Cv*^ mice. Significant genotype effects (*p* < *0.05*) are marked with an asterisk

**Fig. 4:**
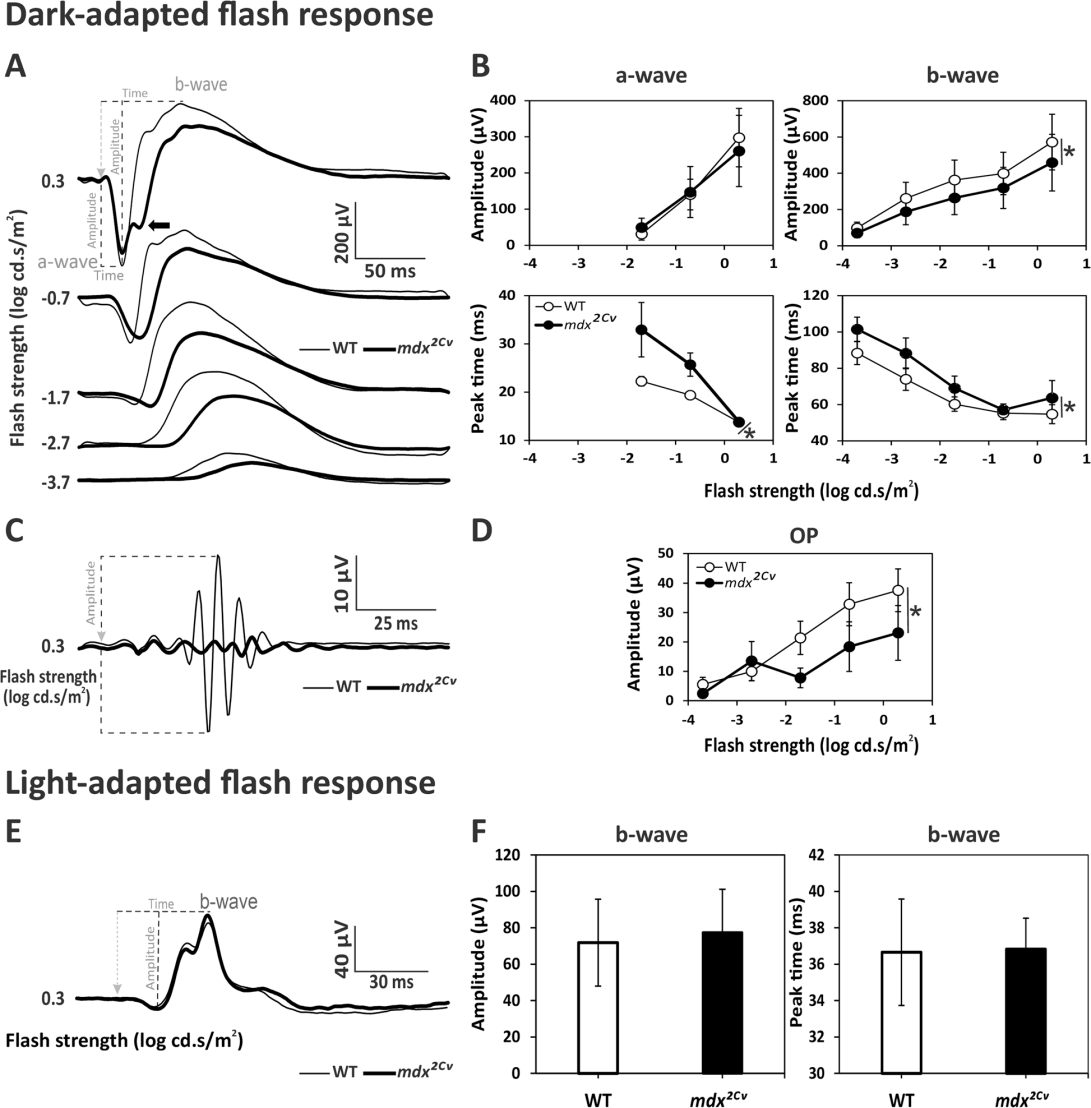
Dark-adapted (DA) and light-adapted (LA) flash ERGs in WT and *mdx*^*2Cv*^ mice. **A** Averaged DA waveforms with OPs removed in WT (thin traces) and *mdx*^*2Cv*^ mice (bold traces). Flash strength and definitions of key components (a-wave, b-wave) are indicated. **B** Mean (± SD) amplitudes (μV; upper plots) and implicit times (ms; lower plots) of DA a- and b-waves as a function of flash strength, in WT (open symbols) and *mdx*^*2Cv*^ mice (filled symbols). **C** Averaged OP traces isolated from the strongest scotopic flash (0.3 log cd.s/m^2^) in WT (thin traces) and *mdx*^*2Cv*^ mice (bold traces). **D** Mean (± SD) amplitude of OPs as a function of flash strength in WT (open symbols) and *mdx*^*2Cv*^ (filled symbols) mice. **E** Averaged LA waveforms of WT (thin traces) and *mdx*^*2Cv*^ mice (bold traces) at 0.3 log cd.s/m^2^ flash strength. **F** Histograms showing the mean (± SD) amplitude and implicit time of the LA b-wave. Dotted lines in A, C and E show the physiological hallmarks used for measurement of amplitudes and/or implicit times; the light-grey arrowhead marks the onset of the stimulus. Recordings made in 32 eyes of 16 WT and 32 eyes of 16 *mdx*^*2Cv*^ mice. Significant genotype effects (*p* < *0.05*) are marked with an asterisk

ERG components were analyzed offline by peak/trough detection, baseline measurements using self-written Matlab® routines (The Mathworks Inc., Natick, Massachusetts, United States) and Excel spreadsheets (Microsoft Office 2021, ©Microsoft Corporation, Redmond, WA, USA). The processing and analyses of ERG signals were previously described [16, 20, 24]. Briefly, the oscillatory potentials (OPs) were isolated from scotopic recordings by a variable digital filter method [34], in which the OPs are separated from the other components in the frequency domain (after Fourier analysis of the responses). ERGs without OPs were used to measure a- and b-wave parameters (see Figs. 2-4A). Isolated OPs (see Figs. 2-4C) were also analyzed. The amplitude of the scotopic a-wave was defined as the difference in μV between the baseline (average of recordings 16 ms before the occurrence of the flash) and the minimum within 50 ms after stimulus onset. The amplitude of the scotopic b-wave was the electric potential difference (in μV) between a-wave trough and the peak of the b-wave. Their implicit times corresponded to the intervals between stimulus onset and the trough of the a-wave and the peak of the b-wave, respectively. The OPs were analyzed in the frequency domain and their amplitudes was defined as the maximal amplitude in the high frequency range of the Fourier spectrum (between 60 and 100 Hz).

The b-waves of the light-adapted flash ERG were measured as described for the dark-adapted condition. All ERG parameters are presented as a function of flash intensity. As previously shown [16, 20, 24], the photopic a-waves were very small and not included in the analyses.

To summarize the differences between the different DMD models, we chose to quantify ERG response change with a single value taking into account the responses at all flash strengths. This allowed us to get single variables representing the main genotype effects. The response amplitudes were first normalized to the response amplitudes obtained in their respective control groups at the same flash strength, in order to express relative changes as the % of WT amplitude (WT mean = 100%). These values were then averaged across flash strengths. For implicit time, the mean value of the respective WT groups was subtracted from each individual value at each flash strength, in order that the mean value of each control group was 0 and the mean of each mouse model was expressed as the difference between mutant and WT values (in ms). The same process was applied to our previously published data from the *mdx52* mouse line lacking Dp427, Dp260, and Dp140 [16] for comparison.

### Statistics

ERG data were expressed as means ± standard deviation (SD) to compare each mouse model to WT littermate controls. Comparisons of averaged data between mouse models were expressed as means ± standard error of the mean (SEM). Detailed statistics for all figures, as well as comparisons of the different WT groups, are shown in Additional file 2: Table S1 and Additional file 2: Table S2, respectively. Both tables also show, side by side, the statistical results obtained when the sample size correspond to the total number of eyes, and after averaging data from the two recorded eyes in each individual mouse. Statistical analysis to determine genotype and group differences were performed with the Jamovi software [The jamovi project (2023); *jamovi* (Version 2.3), Sydney, Australia. Retrieved from https://www.jamovi.org]. Group differences were determined using one or two-way ANOVAs depending on the presence of a within-subject repeated measure (for measurements at different flash strengths). When one-way ANOVAs indicated differences in variance homogeneity (Levene's test), the Welch correction was applied, and a post-hoc Games-Howell test used. For two-way ANOVAs, sphericity was assessed using the Mauchly's test to verify variance homogeneity. When violations were detected, the Greenhouse–Geisser correction was applied for severe violations, and the Huynh–Feldt correction for mild or moderate violations. After correction, multiple comparisons were performed using the Bonferroni post-hoc test. When variance homogeneity was comparable between groups, no corrections were made and the Tukey test was used for post-hoc analysis. The *p* values < *0.05* were considered statistically significant. The traces showing waveforms recorded in response to distinct stimulation protocols were built using the average of all the analyzed eyes in each group.

## Results

In all groups of mice, DMD models and WT littermates, the a-wave was detectable at flash strengths—1.7 log cd.s/m^2^ and above, but not at flash strengths −3.7 and −2.7 log cd.s/m^2^. The amplitudes of the a- and b-waves of the DA flash ERGs increased with increasing flash strength in all genotypes (*p* < *0.001*; Fig. 2-4B; as indicated). Conversely, the implicit times decreased with increasing flash strength in all genotypes (*p* < *0.001*; Fig. 2-4B; as indicated). The OPs at the ascending limb of the b-wave were isolated from original DA ERGs and analyzed in the frequency domain (see Methods section), revealing increased amplitudes with increasing flash intensity across all genotypes (*p* < *0.001*; Fig. 2-4D).

### ERGs in the absence of full-length dystrophin, Dp427

Averaged traces of flash ERG waveforms recorded in Dp427-deficient *mdx* mice (*n* = 34 eyes) and their WT male littermates (*n* = 32 eyes) in DA conditions are shown in Fig. 2A, from −3.7 to 0.3 log cd.s/m^2^ stimulation intensities. The mean amplitudes of the a- and b-waves (Fig. 2B) were slightly but significantly reduced in *mdx* compared with WT mice, on average by about 13% and 17%, respectively (a-waves: *p* = *0.013*; b-waves: *p* = *0.009*), with no significant genotype x flash strength interaction (Additional file 2: Table S1). The implicit times (Fig. 2B) were comparable between genotypes for the a-waves (*p* = *0.581*; Additional file 2: Table S1), while they were slightly reduced by about 3% (corresponding to 2.90 ms) in *mdx* mice for the b-waves (*p* = *0.020*; Additional file 2: Table S1). The OPs are shown in Fig. 2C for the strongest flash strength (0.3 log cd.s/m^2^). As mentioned before, their amplitudes were analyzed in the frequency domain (Fig. 2D) and revealed no difference between *mdx* and WT mice (*p* = *0.575*; Additional file 2: Table S1). The light-adapted (photopic) responses were recorded at 0.3 log cd.s/m^2^ as shown in Fig. 2E. The two genotypes expressed comparable b-wave amplitudes (*p* = *0.395*) and implicit times (*p* = *0.914*).

Adult Dp427-deficient *mdx* mice therefore displayed only marginal changes in DA responses. This was largely confirmed in our study with the *mdx*^*5Cv*^ mouse (*n* = 34 eyes) compared with their WT male littermates (*n* = 32 eyes), presented in Fig. 3, that also selectively lacks Dp427 due to a mutation in another proximal exon of the *dmd* gene. Amplitudes of the a- and b-waves were reduced in the mutant mice compared to WT mice (Fig. 3A-B). The mean amplitudes of the a-waves were statistically comparable between genotypes (*p* = *0.117*; Additional file 2: Table S1), yet there was a significant genotype x flash strength interaction (*p* = *0.041*) highlighting that the difference was larger at the highest intensities. The mean b-wave amplitudes, however, were significantly reduced by 15,58% in *mdx*^*5Cv*^ mice (*p* = *0.007*; Additional file 2: Table S1). Implicit times (Fig. 3B) were comparable between genotypes for both a- and b-waves (*p* = *0.986* *p* = *0.063*, respectively), with no significant genotype x intensity interactions. The OPs (Fig. 3C-D) of *mdx*^*5Cv*^ and WT mice also had comparable amplitudes (Additional file 2: Table S1). The photopic b-waves (Fig. 3E-F) also had comparable amplitudes (*p* = *0.656*) and implicit times (*p* = *0.620*) in the two genotypes. Hence, the only ERG change that was significant in both *mdx* and *mdx*^*5Cv*^ mice was the reduction in the b-wave amplitude, suggesting that alteration of this DA response was the most robust phenotype associated with the absence of Dp427.

For both the *mdx* and *mdx*^*5Cv*^ mouse lines, some differences from WT controls were not significant if the number of animals was considered instead of the number of eyes (i.e., after averaging data from the two recorded eyes in each animal). However, as shown in Additional file 2: Table S1, the same trends are still observed in these two lines where most p values are then comprised between *0.05* and *0.1* instead of being below *0.05*. This statistical comparison therefore still supports the conclusion that Dp427 deficiency alone has a minor impact on ERG, and keeping a larger sample size (number of eyes) allowed us to better capture the small differences that were present in these models. For the other mouse lines (described below), as well as for the comparisons between mouse models, averaging data from the two eyes did not change the statistical comparisons, leading to the same conclusions regarding the genotype-dependent variations.

### Effects of combined Dp427 and Dp260 alterations on ERGs

Characterization of ERG responses in *mdx*^*2Cv*^ mice (*n* = 32 eyes) and their WT male littermates (*n* = 32 eyes) is presented in Fig. 4. Dark-adapted flash responses were clearly smaller in the mutant mice (Fig. 4A). However, there was no main genotype difference for the a-waves amplitudes (*p* = *0.762*), yet there was a significant genotype x flash strength interaction (*p* < *0.003*) reflecting a larger change for responses to stronger flashes (Fig. 4B). In contrast, the mean amplitudes of the b-waves (Fig. 4B) were more strongly and significantly reduced (23.3%) than in *mdx* and *mdx*^*5Cv*^ mice (*p* < *0.001*; Additional file 2: Table S1). The mean implicit times of a- and b-waves (Fig. 4B) were clearly increased in *mdx*^*2Cv*^ mice compared to WT mice, by 31% (5.69 ms) and 14% (9.40 ms), respectively (*p* < *0.001*; Additional file 2: Table S1). Additionally, the scotopic ERG in *mdx*^*2Cv*^ mice exhibited a distinct shoulder on the ascending limb of the b-wave at the highest flash intensity (arrow in Fig. 4A), a feature consistently observed in all mutant individuals but not in WT mice. The OPs extracted from the b-wave dark-adapted flash responses (Fig. 4C) were analyzed in the frequency domain (Fig. 4D), showing a clearly decreased amplitude in *mdx*^*2Cv*^ mice (39,28%) compared with the WT mice (*p* < *0.001*; Additional file 2: Table S1). For the photopic responses (Fig. 4E-F), *mdx*^*2Cv*^ showed no differences when compared with WT littermate mice for both b-wave amplitudes (*p* = *0.356*) and implicit times (*p* = *0.780*).

### ERGs in dystrophin-null retinas

**Fig. 5:**
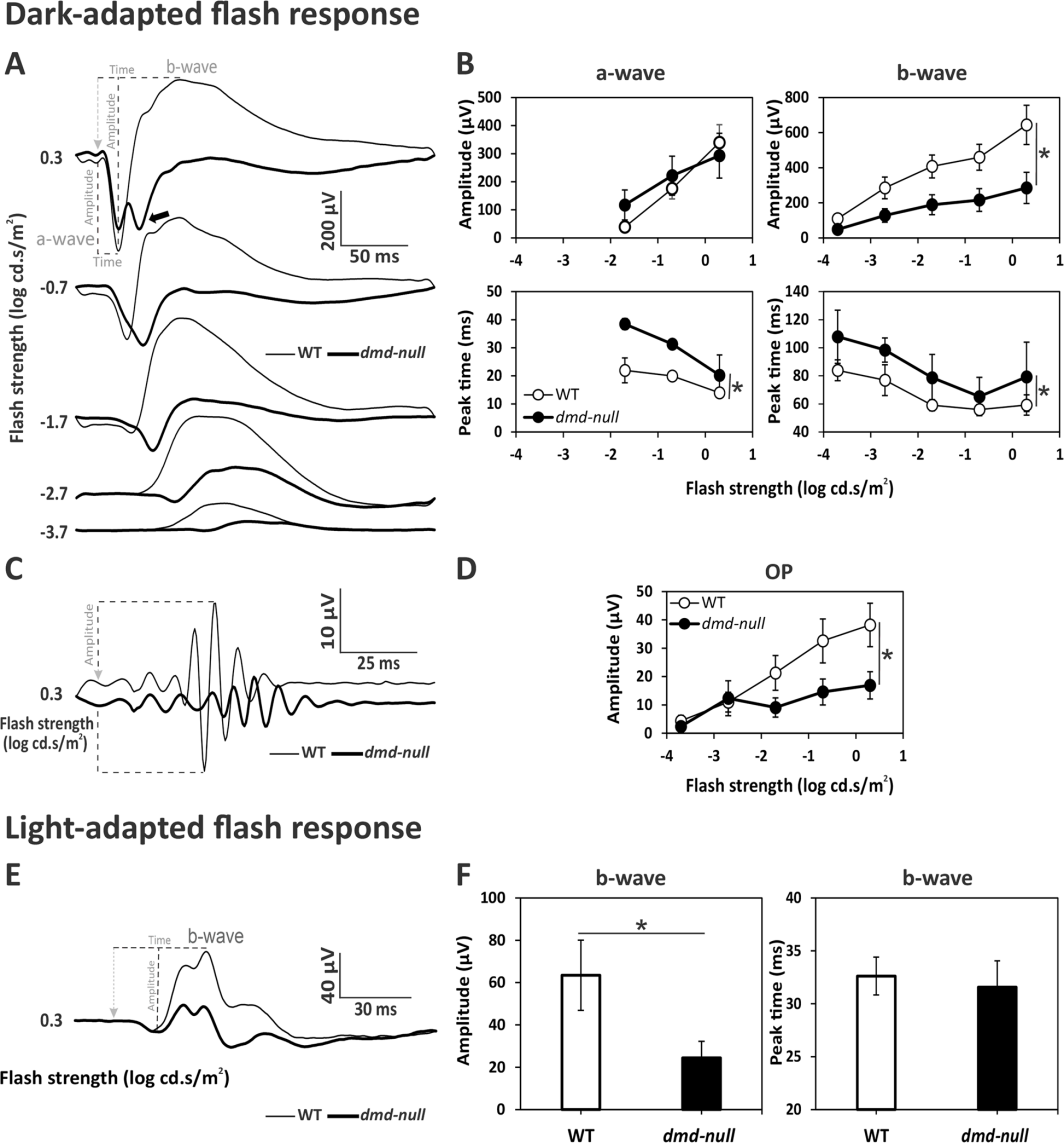
Dark-adapted (DA) and light-adapted (LA) flash ERGs in WT and *dmd-null* mice. **A** Averaged DA waveforms with OPs removed in WT (thin traces) and *dmd-null* (bold traces) mice. Flash strength and definitions of key components (a-wave, b-wave) are indicated. **B** Mean (± SD) amplitudes (μV; upper plots) and implicit times (ms; lower plots) of DA a- and b-waves as a function of flash strength, in WT (open symbols) and *dmd-null* mice (filled symbols). **C** Averaged OP traces isolated from the strongest DA flash (0.3 log cd.s/m^2^) in WT (thin traces) and *dmd-null* mice (bold traces). **D** Mean (± SD) amplitude of OPs as a function of flash strength in WT (open symbols) and *dmd-null* (filled symbols) mice. **E** Averaged LA waveforms of WT (thin traces) and *dmd-null* mice (bold traces) at 0.3 log cd.s/m^2^ flash strength. **F** Histograms showing the mean (± SD) amplitude and implicit time of the LA b-wave. Dotted lines in A, C and E show the physiological hallmarks used for measurement of amplitudes and/or implicit times; the light-grey arrowhead marks the onset of the stimulus. Recordings were made in 24 eyes of 12 WT and 28 eyes of 14 *dmd-null* mice. Significant genotype effects (*p* < *0.05*) are marked with an asterisk

ERG responses of *dmd-null* mice (*n* = 28 eyes) were compared with their WT male littermates (*n* = 24 eyes; Fig. 5). *Dmd-null* mice presented the most severe phenotype among dystrophic models, with a negative ERG at the highest dark-adapted flash response (Fig. 5A). The mean amplitudes of the a-waves (Fig. 5B) were not affected in *dmd-null* mice (*p* = *0.134*), but there was a significant genotype x intensity interaction (*p* < *0.001*) suggesting a larger a-wave amplitude in *dmd-null* mice for the weakest flash where an a-wave could be identified (−1.7log cd.s/m^2^, *p* < *0.001*). In contrast, the b-wave amplitudes were strongly reduced in *dmd-null* mice (by 54.51%; *p* < *0.001*; Additional file 2: Table S1). The mean implicit times of a- and b-waves (Fig. 5B; lower plots) were increased in *dmd-null* mice compared to WT mice, by 61% (11.37 ms) and 28.14% (18.87 ms), respectively (*p* < *0.001*; Additional file 2: Table S1). Furthermore, the scotopic ERG in all *dmd-null* mice exhibited a distinct shoulder on the ascending limb of the b-wave at the highest flash intensity (arrow in Fig. 5A), which was never observed in WT mice, as also shown above for *mdx*^*2Cv*^ mice. This shoulder was more pronounced in the *dmd-null* mice. The OPs extracted from the dark-adapted b-wave (Fig. 5C) were strongly reduced in *dmd-null* mice compared with WT mice, with a significantly reduced amplitudes (by 48.54%; *p* < *0.001*; Additional file 2: Table S1). For the photopic responses (Fig. 5E), the photopic b-wave amplitudes were significantly reduced by 62,90% in *dmd-null* mice compared with WT littermate mice (*p* < *0.001*), while no genotype differences were found for the implicit times (*p* = *0.083*).

### Comparisons between DMD models

**Fig. 6:**
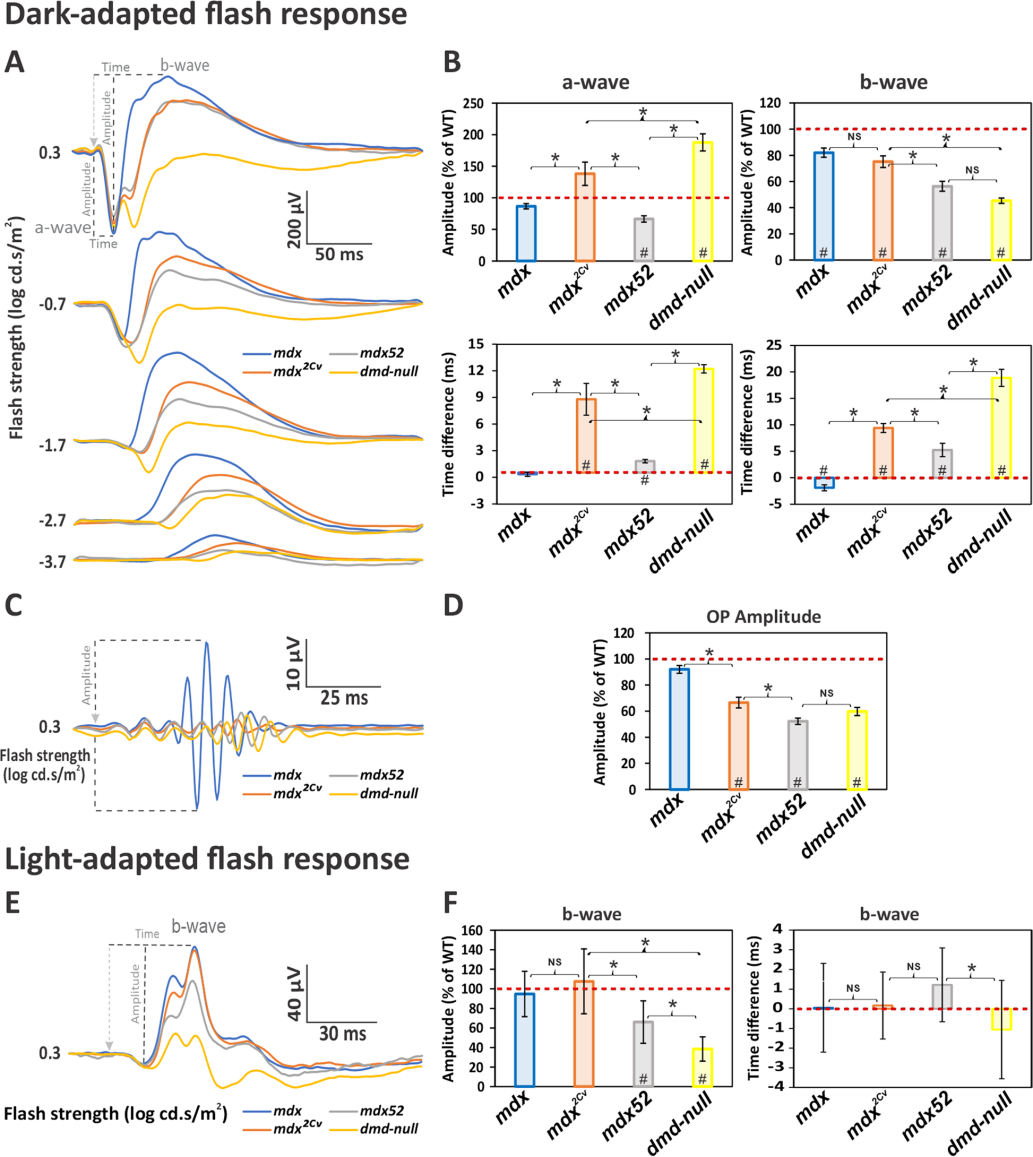
Relative changes of Dark-adapted (DA) and light-adapted (LA) flash ERGs in *DMD* mouse models. **A** Average DA waveforms with OPs removed in *mdx* (blue traces), *mdx*^*2Cv*^ (orange traces), *mdx52* (gray traces) and *dmd-null* mice (yellow traces). Flash strength and definitions of key components (a-wave, b-wave) are indicated. **B** Histograms showing the mean (± SEM) normalized amplitudes (% of WT; upper plots) and the difference between WT and mutant implicit times (ms; lower plots) for DA a- and b-waves, in *mdx* (blue), *mdx*^*2Cv*^ (orange), *mdx52* (gray) and *dmd-null* (yellow) mice. **C** Averaged OP traces isolated from the strongest scotopic flash (0.3 log cd.s/m^2^) in *mdx* (blue), *mdx*^*2Cv*^ (orange), *mdx52* (gray) and *dmd-null* mice (yellow). **D** Histograms showing the mean (± SEM) normalized amplitude of OPs in *mdx* (blue), *mdx*^*2Cv*^ (orange), *mdx52* (gray) and *dmd-null* (yellow) mice. **E** Averaged LA waveforms of *mdx* (blue), *mdx*^*2Cv*^ (orange), *mdx52* (gray) and *dmd-null* (yellow). **F** Histograms showing the mean (± SD) amplitude and implicit time of the LA b-wave. The red dotted lines on histograms represents the control (WT). Dotted lines in A, C and E show the physiological hallmarks used for measurement of amplitudes and/or implicit times; the light-grey arrowhead marks the onset of the stimulus. Recordings were made in 34 eyes of *mdx*, 34 eyes of *mdx*^*2Cv*^, 20 eyes of *mdx52* and 28 eyes of *dmd-null* mice. Significant differences from WT littermate means are marked with a # (*p* < *0.05*) in the bar plot; significant differences between DMD mouse lines are marked with an asterisk (*p* < *0.05*). NS: not significant

In Fig. 6, we summarize and compare the ERG defects in the different DMD mouse models, by presenting the ERG changes relative to their respective WT littermate control group averaged across all flash strengths (see details in the Methods section). Some statistical differences were detected between WT groups (Additional file 2: Table S2), which pointed to inter-experiment variability and warranted a normalization of mutant data to their respective WT littermate controls. The *mdx* line was used to represent the relative changes induced by a selective loss of Dp427, because this is the most widely used Dp427-deficient mouse model. As mentioned above the ERG changes in the *mdx*^*5Cv*^ mice were very similar to those displayed by *mdx* mice, and the use of *mdx*^*5Cv*^ mice instead of *mdx* mice would have led to identical conclusions (Additional file 2: Table S3). We included the results of our previously published study with *mdx52* mice lacking Dp427, Dp260 and Dp140, which were generated under identical conditions (raw data are shown in Fig. 2 of this publication: [16] and are available in Liber et al. (2026) [35]). Normalized amplitudes in Fig. 6 are expressed as % of WT (WT mean = 100%; dotted red lines), while the implicit times are expressed as the difference in ms between mutants and WT mice (WT mean = 0 ms; red dotted lines). Averaged waveforms are also shown for each parameter. Figure 6A shows that DA flash responses clearly differed between DMD mouse models at all stimulus intensities. Figure 6B shows the mean difference of ERG response amplitudes between DMD mouse models and WT littermate in DA conditions. There was a significant genotype effect for the a-wave amplitudes (*p* < *0.001*; Additional file 2: Table S1). Post hoc tests revealed that only *mdx52* (*p* < *0.001*) and *dmd-null* mice (*p* < *0.001*) displayed significant changes as compared to their WT littermates. Amplitudes were smaller in *mdx* compared to *mdx*^*2Cv*^ mice (*p* < *0.05*), smaller in *mdx52* than in *mdx*^*2Cv*^ mice (*p* < *0.001*), while they were larger in *dmd-null* mice compared to *mdx52* mice (*p* < *0.001*) and *mdx*^*2Cv*^ mice (*p* < *0.01*). There was also a significant genotype effect for the b-wave amplitudes (*p* < *0.001*; Fig. 6B). The b-wave amplitudes in all DMD models were significantly smaller compared to their respective WT littermates (*p* < *0.01*). Comparisons between the DMD models revealed no differences between *mdx* and *mdx*^*2Cv*^ (*p* = *0.624*), while both *mdx52* (*p* < *0.02*) and *dmd-*null mice (*p* < *0.001*) had smaller b-wave amplitudes compared to *mdx*^*2Cv*^ mice. Interestingly, b-wave amplitudes were comparable between *mdx52* and *dmd-null* mice (*p* = *0.075*). To summarize, there was a progressive decrease in b-wave amplitudes, particularly emphasized by the loss of Dp140, but no further decrease when Dp71 was lost.

For the implicit times (Fig. 6B, bottom plots), there was a significant genotype effect for the a-waves (*p* < *0.001*), with significant increases for all DMD models (*p* < *0.001*) except *mdx* mice (*p* > *0.8*) when compared to their respective WT littermates. This indicates that the loss of Dp260 increases implicit times of the a-wave. However, implicit times were shorter in *mdx52* mice compared with *mdx*^*2Cv*^ and *dmd-null* mice (both *p* < *0.001*), while they were longer in *dmd-null* mice (*p* < *0.001*) compared to all other DMD mouse models, suggesting a larger impact of Dp71 loss. The b-wave implicit times also showed a significant genotype effect (*p* < *0.001*). The b-wave implicit times were longer for all models (*p* < *0.001*) except for *mdx* mice that showed a slight reduction (*p* < *0.05*) as compared to their respective WT littermates. Implicit times were significantly longer in *dmd-null* mice than in *mdx*^*2Cv*^ and *mdx52* mice (*p* < *0.001*). This, again, suggests that Dp260 loss increases b-wave implicit times, a phenotype that is further aggravated when all dystrophins are absent.

Figure 6C shows that the OP average waveforms extracted from the maximum scotopic response are unaffected in *mdx* mice, but drastically reduced in all other DMD mouse models. Normalized OP amplitudes (Fig. 6D) were significantly different among mouse models (*p* < *0.001*) and post hoc analyses confirmed that the Dp427-deficient *mdx* mouse was the only line showing unaffected OP responses compared to WT littermates (*mdx*: *p* = *0.105*; other models: *p* < *0.001*). In the comparisons between DMD models, we detected a small aggravation in *mdx52* mice compared with *mdx*^*2Cv*^ mice (*p* = *0.021*). This indicates that the loss of Dp260 is responsible for a significant reduction in OPs amplitude, which is slightly worsened with the loss of other dystrophins.

Figure 6E presents the average waveforms of LA (photopic) responses in all DMD mouse models, showing a decreased amplitude for *mdx52* and *dmd-null* mice. The normalized photopic b-wave amplitudes (Fig. 6F; as indicated) were different among mouse models (*p* < *0.001*). When compared with their respective control groups, significant reductions were detected only for *mdx52* (*p* < *0.01*) and *dmd-null* mice (*p* < *0.001*). However, amplitudes were smaller in *mdx52* than in *mdx*^*2Cv*^ mice (*p* < *0.001*), and even shorter in *dmd-null* mice compared with *mdx52* mice (*p* < *0.001*). These results demonstrate that the absence of Dp427 and Dp260 does not affect the amplitude of LA flash ERG, which are, however, progressively decreased with the additional loss of Dp140, or of Dp140 and Dp71. The LA b-waves implicit times (Fig. 6F, right plot) were comparable between each mouse models and its respective WT littermate group (*p* > *0.08* for all models). However, comparisons among the mouse models detected a significant difference between *mdx52* and *dmd-null* mice, with *dmd-null* mice showing shorter implicit times compared to *mdx52* mice (*p* < *0.01*). Indeed, only the *dmd-null* mice showed a mean decrease in the implicit time.

## Discussion

Retinal abnormalities were identified several years ago in patients with DMD [26, 36, 37] and subsequently claimed to be genotype-dependent [11, 12, 15]. A genotype-dependent retinal dysfunction, as measured with ERGs, was also found to be present in mouse models for DMD. Normal or slightly affected ERGs were previously reported in different *mdx* strains, except for the *mdx*^*3Cv*^ showing evident retinal dysfunction [13], as subsequently confirmed by our group [24]. This suggested that only the simultaneous alteration of all retinal dystrophins would cause significant ERG changes mimicking the human phenotype [13]. The present results show that, in fact, all individual retinal dystrophins (Dp427, Dp260, Dp140, Dp71) influence retinal electrophysiology. Moreover, we highlight genotype–phenotype correlations linking mutation location, cumulative loss of several dystrophins, and phenotype severity, as well as specific functional outcomes involving the distinct dystrophins. The ERGs of the Dp427-deficient *mdx* and *mdx*^*5Cv*^ mouse models holding mutations in exon 23 and exon 10, respectively, were initially reported to be normal [13, 26], although they present muscular and behavioral limitations caused by DMD. More recently, Bucher et al. have demonstrated that the retinal alterations in *mdx* mice are indeed minor, at least in young adult animals (12–16 weeks old), but deteriorate with aging (15- 18 months old) [25]. Our present results are in agreement with these results by indeed showing mild alterations of the ERG in young adult *mdx* mice, limited to slightly reduced DA (rod-dominated) a- and b-waves. ERGs from another DMD model (*mdx*^*5Cv*^) that also lack Dp427 confirm these mild ERG changes.

Dp427 has been detected in the outer plexiform layer (OPL) of the mouse retina [14]. In addition, Dp427 mRNA was found in the OPL with an apparent enrichment in the ONL [8], which we in part confirmed by detection of the mRNA of the Dp427p isoform in purified photoreceptors [5]. The fact that only a mild retinal phenotype is observed in the Dp427-deficient *mdx* mouse suggests a minor role for Dp427 in photoreceptor function. This effect could be either a direct influence of Dp427 on retinal physiology or indirect through Dp427-induced cardiorespiratory, muscular, metabolic and/or inflammatory alterations [38]. Alternatively, one may hypothesize presence of systemic compensatory mechanisms in the retina that might not take place in the brain. Indeed, *mdx* mice display a range of brain-related behavioral phenotypes, from emotional and social behavior disturbances to learning and memory deficits [9]. A putative contribution of systemic alterations could be investigated in future preclinical studies, by testing ERG in *mdx* mice under a treatment restoring muscle integrity. Apart from concluding on the role of Dp427, the evaluation of the *mdx* model is extremely important for deciphering the roles that the other dystrophin isoforms play in retinal electrophysiology as discussed in the following sections.

The *mdx*^*2Cv*^ model lacking the retinal dystrophin Dp260 in addition to Dp427, due to the genetic alteration in intron 42, showed more pronounced ERG changes than the *mdx* mouse. Importantly, both models share identical genetic background (C57BL/6) and were examined at the same age (3–4 months old). As a result, the affected ERG components observed in *mdx*^*2Cv*^, but not in *mdx*/*mdx*^*5Cv*^ mice, allows a speculation about the specific functional roles of Dp260 in the mouse retina. Dp260 is found in the invaginating ribbon synapse of the photoreceptors that connect to depolarizing On-bipolar cells [8, 39, 40]. The absence of Dp260 was associated with β-dystroglycan delocalization in OPL, due to a disruption of the dystrophin-dystroglycan complex [14].

The ERG components specifically affected in *mdx*^*2Cv*^ mice suggest that Dp260 is involved in multiple cellular mechanisms. Indeed, both slow (a-wave/b-wave) and fast (oscillatory potentials) ERG components were reduced in amplitude in *mdx*^*2Cv*^ mice under dark-adapted condition. The alteration of the oscillatory potentials (OPs) points to a disturbance in the inner retina, possibly of the amacrine and/or ganglion cells which are claimed to be the origin of the OPs [41]. It could be either a consequence of the disturbed input from photoreceptor to bipolar cell or of a direct effect of Dp260 alteration in the inner retina. Both hypotheses might be relevant, as the Dp260 protein has been detected at the ribbon synapse, while its mRNA was also detected in the INL that contains bipolar, amacrine and horizontal cells [1, 8]. Interestingly, the *mdx*^*2Cv*^ model showed a “shoulder” on the ascending limb of the b-wave at the highest flash strengths (see Fig. 4A arrow). This has also been reported in mice with ribbon synapse alteration and congenital stationary night blindness due to CACNA1F gene mutations [42], and in *mdx52* mice (see below). This feature was neither observed in WT mice and *mdx* mice lacking only Dp427, nor in Dp71-null mice lacking only Dp71 [20]. The preserved ERGs under light-adapted conditions indicate that Dp260 does not have a major function in cone-driven pathways.

The absence of Dp260 was associated with β-dystroglycan delocalization in OPL, due to a disruption of the dystrophin-dystroglycan complex [14]. Dystroglycan is a core transmembrane component of the dystrophin-associated complex in various cell types, from skeletal muscle fibers to central inhibitory synapses. Bridging extracellular matrix proteins to cytosolic and cytoskeletal elements, dystroglycan contributes to cell signaling and clustering of different receptors and channels at synaptic membranes. In retina, dystroglycan normally binds the extracellular-matrix protein Pikachurin, which is essential for bipolar dendrite connection to photoreceptor ribbon synapse, clustering of the G protein-coupled postsynaptic receptor GPR179 to ON-bipolar cell dendritic terminals, metabotropic signaling cascade, and signal transmission from rods to rod-bipolar cells [43–45]. Therefore, a destabilization of this molecular mechanism alters ribbon-synapse development, architecture and function, which likely underlies the critical role of Dp260 in retinal electrophysiology.

To evaluate the specific role of Dp140 play in retinal electrophysiology, the results from *mdx*^*2Cv*^ mice lacking Dp427 and Dp260, were compared to previous results from the *mdx52* mouse model of DMD [16]. The *mdx52* model holds a deletion of exon 52 and, therefore, lacks Dp427, Dp260 and Dp140 [46, 47]. Here we show that several ERG parameters are more strongly affected in *mdx52* than in *mdx*^*2Cv*^ mice, suggesting that Dp140 indeed affects the ERG phenotype of DMD mouse models. Specifically, the a-wave amplitudes of the DA ERG were not affected in *mdx*^*2Cv*^, while they were significantly reduced, and implicit times prolonged, in *mdx52*. The dark-adapted b-waves were delayed in both *mdx*^*2Cv*^ and *mdx52*, and the amplitudes were more strongly reduced in *mdx52* mice. The OP amplitude was also more strongly reduced in *mdx52* mice. However, the light-adapted b-wave amplitude was significantly reduced in *mdx52* but not in *mdx*^*2Cv*^ mice. Our results therefore strongly support the notion that Dp140 plays specific roles in cone and rod driven retinal electrophysiology.

The localization of Dp140 in the retina is not well understood. Although Dp140 mRNA has been detected in the ONL, INL and the photoreceptors, specific antibodies that can confirm protein expression at the cellular level are lacking [1, 5]. Our recent RNA-Seq analysis of purified photoreceptors suggests expression in the cones of the mouse retina [5], what could explain the cone-driven (LA) ERG alterations.

It has been demonstrated that the lack of Dp140 in the brain is associated with worsening of the cognitive dysfunction and neuropsychiatric outcomes found in approximately 30% of the DMD patients [48, 49]. In addition, recent reports have emphasized the increased severity of emotional disturbances caused by Dp140 alterations in mice [50, 51]. Our present results also confirm that Dp140 deficiency affects other parts of the CNS. Therefore, to overcome cognitive impairment and behavioral disorders in DMD patients, therapeutic solutions should include rescuing Dp140 expression in addition to Dp427 in the brain. The ERGs may be an adequate biomarker to evaluate the benefit of restoring Dp140 function in the CNS.

The effects of a loss of all dystrophins, including Dp71, on retinal electrophysiology were analyzed in *dmd-null* mice. This revealed the most severe ERG phenotype among all mouse lines: The DA ERG was negative (i.e. the b-wave was smaller than the a-wave) and the peaks were more strongly delayed for both a- and b-waves compared with *mdx52* mice that still express Dp71. These results suggest that the transmission between rods and bipolar cells (again particularly of the On-type) are strongly affected. The OPs were reduced but comparable to those recorded in *mdx52* mice, indicating that the additional loss of Dp71 does not affect the inner retina (unless its input is so strongly disturbed that an effect on its physiology is not feasible anymore). LA b-waves were below 50% of WT amplitudes and significantly smaller than those recorded in the other DMD models. Moreover, although there were no significant differences when mouse models were compared to their respective WT littermates, the *dmd-null* mouse was the only model showing significantly shorter implicit times under photopic condition compared to the other mouse models. These results demonstrate that cones and their connections are additionally affected by the absence of functional Dp71. In the Dp71-null mouse, which displays a selective loss of Dp71, ERG b-wave amplitudes were reduced under both dark- and light-adapted conditions. Moreover, sine-wave flicker responses at low temporal frequencies and photopic On-responses were altered, pointing to defective cone processing. Importantly, since Dp71 is selectively expressed in retinal Müller glial cells and astrocytes, the ERG alteration observed in this model cannot be attributed altered photoreceptor to bipolar cell synaptic transmission. Instead, it results from an imbalanced ionic homeostasis caused by Müller glial-cell dysfunction [20]. Indeed, Dp71contributes to the proper distribution of the inward rectifying potassium Kir4.1 channels and the Aquaporin-4 water transport channels. The selective loss of Dp71 in the mouse retina is associated with a deregulation of retinal water and potassium homeostasis, altered retinal blood barrier and reduced DA and LA ERG b-waves [19, 20, 52, 53]. Based on these findings, it seems reasonable to speculate that the loss of Dp71, combined with the absence of other dystrophins, leads to a more severe retinal phenotype because of these additional alterations of ionic homeostasis and retinal blood barrier.

Our comparative study of several mouse models holding distinct mutations is representative of the distinct subgroups of patients with different mutation locations and number of affected dystrophins. A selective loss of the full-length dystrophin protein Dp427 is found in a significant portion of DMD patients due to a hotspot mutation around exons 2–22 of the DMD gene [54]. These DMD patients holding out-of-frame genetic alterations upstream exon 30 of the *DMD* gene, i.e. before the promotor region of Dp260, lack Dp427 only. ERGs obtained from these patients were reported to be slightly affected and clearly more preserved than the ERG of patients holding more distal genetic alterations and lacking multiple dystrophins [12, 15]. Thus, our results in the Dp427-deficient *mdx* and *mdx*^*5Cv*^ mice are in line with the mild retinal outcome reported in patients with a similar biochemical profile.

The loss of Dp427, Dp260 and Dp140 in DMD patients with genetic alterations in the central mutation hotspot (exons 45–55) results in electronegative ERGs [15], asymmetric ERG alterations to On and Off responses [55] and changes in ERGs to heterochromatic sinewave modulation [56]. The contribution of Dp140 to the ERG phenotypes in DMD patients has not been explored. To our knowledge there are no studies comparing ERGs from patients with genetic alterations affecting Dp427 and Dp260 with ERGs from patients with genetic alterations affecting Dp427, Dp260, and Dp140. Consequently, it is currently difficult to discriminate the retinal alterations respectively caused by the loss of Dp260 and Dp140 in patients. Our comparison of *mdx*^*2Cv*^ and *mdx52* mice provides new insights on this question: We show that Dp140 deficiency worsens the reduction of flash ERG amplitudes observed in *mdx*^*2Cv*^ mice, and specifically reduces the b-wave amplitudes in light-adapted conditions, suggesting a key role in influencing cone-driven signal transmission. Although evidence for cone dysfunction in DMD was reported in a single study [56], our results suggest that future investigations should place a focus on DMD patients lacking Dp140.

In DMD patients, investigating the effects of Dp71 deficiency is more challenging because this condition is only encountered when distal mutations prevent expression of all dystrophins. This is observed in less than 10% of the DMD patients [57]. Two DMD patients with this type of genetic alteration (downstream of exon 63) were reported to have a more severe ERG phenotype, compared to patients with preserved Dp71 expression [12]. The severe phenotype we report here in *dmd-null* mice is in line with this observation.

Finally, DMD patients may face a higher risk of visual impairments with age, warranting further studies of retinal comorbidities in this disease. However, it is worth noting that it is still unclear whether this is directly linked to the genetic alteration of retinal dystrophins, or a secondary effect of treatments, inflammation or age-related cardiorespiratory decline [1]. Variations in the severity of the myopathy have also been reported in mice, which have been attributed to the genetic background of the parental strain [58], age-dependent decline in mice over one year old [59], or associated with the loss of brain dystrophin isoforms [50, 51, 60]. Dystrophin-deficient models may thus follow different systemic trajectories and therefore age-matched cohorts may not represent identical functional “stages” across genotypes. However, the mice in the present study were all on the same genetic background and tested at 3–4 months old, a young-adult time point after retinal maturation and after the early acute degeneration/necrosis peak typical of *mdx* skeletal muscle (∼3–4 weeks), with partial stabilization by ~ 8 week. Major retinal deterioration of ERG responses have been observed in old *mdx* mice (15–18 months old), while ERGs in younger ones (3–4 months) are robust and less confounded by early developmental transitions [25]. While our identical recording conditions mitigate confounding, stage-dependent systemic factors could still indirectly influence ERG outcomes [61]. Importantly, however, the mouse lines also differ by CNS isoform deficiency, which may further shift overall functional status and phenotype severity. ERG phenotypes are shaped by retina-relevant dystrophin isoforms (notably Dp260 and Dp71): Dp260 disruption produces ERG abnormalities including delayed b-wave timing, and Dp71 shows distinct retinal localization and functional contribution. Consistent with this isoform framework, mouse and clinical genotype–ERG studies link more “electronegative” scotopic ERGs to genotypes predicted to disrupt Dp260 (with stronger effects when Dp71 is also affected). Longitudinal ERG across ages, combined with retinal molecular/structural analyses, will be valuable for defining genotype-specific temporal dynamics and for separating systemic stage effects from retina-intrinsic mechanisms.

## Conclusions

Based on our ERG screening in DMD mouse models, retinal dystrophins seem to play specific physiological roles that may be complementary, their absence affecting distinct cell types and pathways. The distinct ERG defects reported in the different mouse models are in agreement with our current knowledge on the specific expression of each dystrophin in the retina. The statistical comparison of mouse models lacking different numbers of dystrophin-gene products clearly demonstrate that distal mutations affecting several dystrophins simultaneously lead to more severe ERG phenotypes, similar to those reported in patients. Interestingly, this worsening of retinal alterations with more distal mutations parallels the impact of proximal-to-distal mutations and cumulative loss of dystrophins on the intelligence quotient in DMD patients. Importantly, the specific ERG profiles resulting from these distinct mutations suggest that the ERG may serve as a relevant, precise and non-invasive biomarker to stratify patients in subgroups displaying different degrees of severity for CNS-related dysfunctions. This might be particularly relevant for patients with mutations involving exon 45 to exon 50, for whom it is difficult to predict from the genotype the effects on Dp140 expression, as the Dp140 promoter is in intron 44 but its translation start site in exon 51.

This hypothesis that the ERG phenotype may reflect broader genotype-dependent CNS dysfunction should be tested in future longitudinal studies correlating retinal and brain-dependent (electrophysiological, behavioral) parameters in DMD mouse models and/or in DMD patients with comparable mutation profiles. This would strengthen the predictive value of ERG and determine whether it can be considered as a biomarker for tracking disease progression and treatment responses, as in other neurodegenerative, neurological and psychiatric disorders [62–66]. Emerging therapies for DMD, based on ectopic re-expression of dystrophins with adeno-associated (AAV) vectors or gene-correction strategies with antisense oligonucleotides, provide hope to rescue expression and function of the different dystrophin isoforms in the CNS [9]. We have previously shown that intra-vitreal injections of an AAV-Dp71 vector could fully rescue the ERG phenotype in Dp71-null mice [20], indicating that ERG defects in DMD mouse models may be responsive to gene therapies. Likewise, antisense oligonucleotides, used to skip mutated exons and rescue expression of the other isoforms, may enable functional rescue in the other DMD mouse lines. Longitudinal ERG assessments during systemic (intravenous) therapeutic interventions, complemented by quantifications of dystrophin re-expression in muscle, brain and retinal tissue, may be an ultimate step to validate the predictive value of ERGs for DMD.

## Supplementary Information

Additional file 1.

Additional file 2.

## Data Availability

The primary data for this study are available online under a Creative Commons license: Liber, A., Barboni, M., Aoki, Y., Kremers, J., Vaillend, C. Electroretinogram defects in mouse models of Duchenne muscular dystrophy. G-Node https://doi.gin.g-node.org/10.12751/g-node.91g71z/ (2026).

## Abbreviations

ANOVA: Analysis of Variance
ARVO: Association for Research in Vision and Ophthalmology
CACNA1F: Calcium Voltage-gated Channel Subunit Alpha1 F
cd.s/m^2^: Candelas per square meter
CNRS: Centre national de la recherche Scientifique (National Center for Scientific Research)
CNS: Central Nervous System
DA: Dark-adapted
DMD: Duchenne Muscular Dystrophy
DNA: Deoxyribonucleic Acid
Dp: Dystrophin
ERG: Electroretinograms
GPR179: G protein-coupled receptor 179
Hz: Hertz
INL: Inner Nuclear Layer
Kir4.1: Inwardly Rectifying Potassium Channel 4.1
LA: Light-adapted
MGC: Müller Glial Cells
mRNA: Messenger Ribonucleic Acid
ONL: Outer Nuclear Layer
OP: Oscillatory Potentials
OPL: Outer Plexiform Layer
PCR: Polymerase Chain Reaction
RNA-Seq: Ribonucleic Acid Sequencing
SEM: Standard Error of the Mean
SD: Standard Deviation
WT: Wild-type
