# Supplementary figures and images for: Dystrophin-gene mutation location influences severity of electroretinogram defects in mouse models of Duchenne muscular dystrophy
Source: BMC Med. 2026 Apr 25;24:271. doi: 10.1186/s12916-026-04873-1 (PMC13123233; doi:10.1186/s12916-026-04873-1)

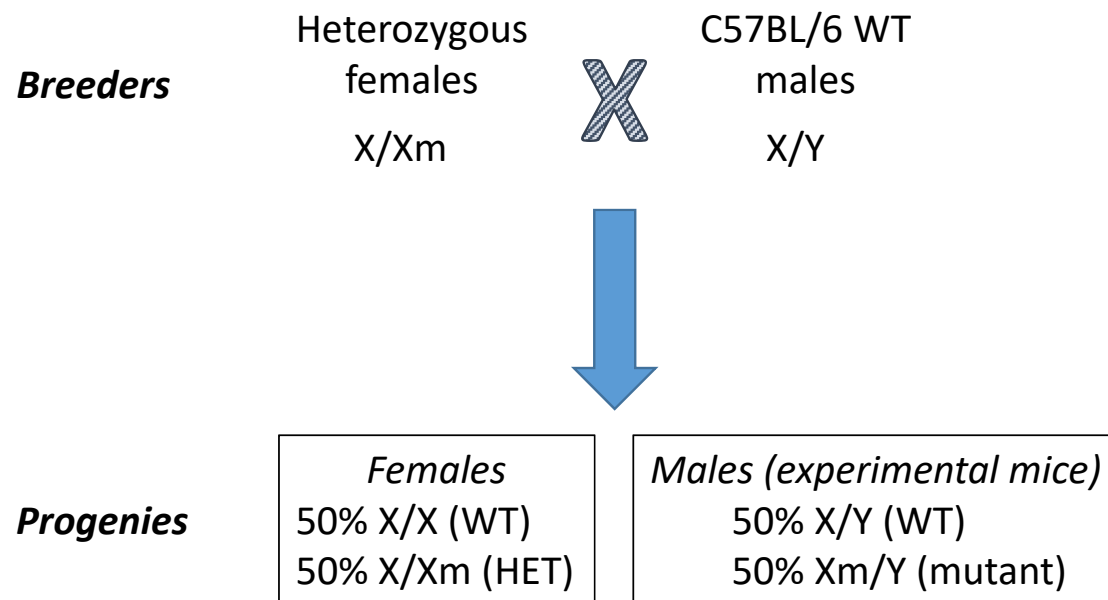

Supplement: Supplementary file 1 — Additional file 1. [file 12916_2026_4873_MOESM1_ESM.pdf]
