# Supplementary material for: Dystrophin-gene mutation location influences severity of electroretinogram defects in mouse models of Duchenne muscular dystrophy
Source: BMC Med. 2026 Apr 25;24:271. doi: 10.1186/s12916-026-04873-1 (PMC13123233; doi:10.1186/s12916-026-04873-1)
Supplement: Supplementary file 2 — Additional file 2. [file 12916_2026_4873_MOESM2_ESM.pdf]

N = Number of eyes

Mouse model	Figure	Condition	Wave	Parameter	Genotype, interaction	F	p
mdx	Fig. 1B	Dark-adapted	a-wave	amplitude	Genotype	F(1,56) = 6.66	0.013
					Genotype x flash	F(1.37,76.51) = 3.18	0.065
			b-wave	amplitude	Genotype	F(1,64) = 7.29	0.009
					Genotype x flash	F (1.47,94.16) = 2.78	0.083
			a-wave	implicit time	Genotype	F(1,58) = 0.307	0.581
					Genotype x flash	F(1.49,86.39) = 0.347	0.644
			b-wave	implicit time	Genotype	F(1,64) =5.72	0.02
					Genotype x flash	F(3.44,220.34) = 4.13	0.005
	Fig. 1D		OPs	amplitude	Genotype	F (1,64) =0.318	0.575
					Genotype x flash	F (1.98,126.64) = 0.884	0.415
	Fig. 1F	Light-adapted	b-wave	amplitude	Genotype	F (1,64) = 0.7332	0.395
				implicit time	Genotype	F (1,64) = 0.0119	0.914
mdx^{5Cv}	Fig. 2B	Dark-adapted	a-wave	amplitude	Genotype	F (1,59) = 2.53	0.117
					Genotype x flash	F (1.19,69.95) = 4.07	0.041
			b-wave	amplitude	Genotype	F (1,64) = 7.72	0.007
					Genotype x flash	F (1.57,100.47) = 4.22	0.025
			a-wave	implicit time	Genotype	F (1,62) = 0.000299	0.986
					Genotype x flash	F (1.59,98.29) = 0.00126	0.996
			b-wave	implicit time	Genotype	F (1,64) = 3.58	0.063
					Genotype x flash	F (3.43,219.41) = 0.874	0.467
	Fig. 2D		OPs	amplitude	Genotype	F (1,64) = 3.41	0.069
					Genotype x flash	F (2.72,173.99) = 1.03	0.376
	Fig. 2F	Light-adapted	b-wave	amplitude	Genotype	F (1,64) = 0.200	0.656
				implicit time	Genotype	F (1,64) = 0.248	0.62
mdx^{2Cv}	Fig. 3B	Dark-adapted	a-wave	amplitude	Genotype	F (1,62) = 0.0929	0.762
					Genotype x flash	F (1.31,81.44) = 7.78	0.003
			b-wave	amplitude	Genotype	F (1,62) = 12.0	<0.001
					Genotype x flash	F (1.57,97.31) =4.96	0.014
			a-wave	implicit time	Genotype	F(1,62) = 179	<0.001
					Genotype x flash	F (1.24,76.94) = 82.3	<0.001
			b-wave	implicit time	Genotype	F(1,62) = 87.5	<0.001
					Genotype x flash	F (4,248) = 13.2	<0.001
	Fig. 3D		OPs	amplitude	Genotype	F (1,62) = 56.8	<0.001
					Genotype x flash	F (2.20,136.53) = 51.4	<0.001
	Fig. 3F	Light-adapted	b-wave	amplitude	Genotype	F (1,62) = 0.8660	0.356
				implicit time	Genotype	F (1,62) = 0.0787	0.78
dmd-null	Fig. 4B	Dark-adapted	a-wave	amplitude	Genotype	F (1,47) = 2.33	0.134
					Genotype x flash	F (1.50,70.49) = 70.4	<0.001
			b-wave	amplitude	Genotype	F (1,50) = 199	<0.001
					Genotype x flash	F (1.67,83.25) = 82.1	<0.001
			a-wave	implicit time	Genotype	F(1,49) = 358	<0.001
					Genotype x flash	F (1.35,66.06) = 24.0	<0.001
			b-wave	implicit time	Genotype	F (1,50) = 92.3	<0.001
					Genotype x flash	F (3.05,152,44) = 2.42	0.067
	Fig. 4D		OPs	amplitude	Genotype	F (1,50) = 96.2	<0.001
					Genotype x flash	F (2.59,129.25) = 87.3	<0.001
	Fig. 4F	Light-adapted	b-wave	amplitude	Genotype	F (1,31.7) = 112.02	<0.001
				implicit time	Genotype	F (1,48.5) = 3.12	0.083
All models compared	Fig. 5B	Dark-adapted	a-wave	amplitude	Genotype	F (3,55.2) = 24.6	<0.001
			b-wave	amplitude	Genotype	F (3,55.3) = 32.2	<0.001
			a-wave	implicit time	Genotype	F (3,52.1) = 223	<0.001
			b-wave	implicit time	Genotype	F (3,51.4) = 75.5	<0.001
	Fig. 5D		OPs	amplitude	Genotype	F (3,60.2) = 36.2	<0.001
	Fig. 5F	Light-adapted	b-wave	amplitude	Genotype	F (3,54.2) = 74.5	<0.001
				implicit time	Genotype	F (3,56.3) = 4.25	0,009

N= Number of animals (Average of the two eyes per individual)

Mouse model	Figure	Condition	Wave	Parameter	Genotype, interaction	F	p
mdx vs WT	Fig. 1B	Dark-adapted	a-wave	amplitude	Genotype	F(1,27)=3.50	0,072
					Genotype x flash	F(1.39,37.46)=1.67	0,206
			b-wave	amplitude	Genotype	F(1.31)=3.82	0,060
					Genotype x flash	F(1.45,44.98)=1.50	0,233
			a-wave	implicit time	Genotype	F(1,27)=3.61	0,068
					Genotype x flash	F(1.15,30.99)=2.48	0,122
			b-wave	implicit time	Genotype	F(1,31)=3.28	0,080
					Genotype x flash	F(3.34,103.60)=2.61	0,049
	Fig. 1D		OPs	amplitude	Genotype	F(1,31)=0.179	0,675
					Genotype x flash	F(1.80,55.85)=0.532	0,572
	Fig. 1F	Light-adapted	b-wave	amplitude	Genotype	F(1,31)=0.425	0,519
				implicit time	Genotype	F(1,31)=0.00740	0,932
mdx^{5Cv} *vs WT*	Fig. 2B	Dark-adapted	a-wave	amplitude	Genotype	F(1,28)=1.39	0,248
					Genotype x flash	F(1.21,34.01)=2.11	0,153
			b-wave	amplitude	Genotype	F(1,30)=3.54	0,070
					Genotype x flash	F(1.49,44.58)=1.81	0,182
			a-wave	implicit time	Genotype	F(1,30)=0.236	0,630
					Genotype x flash	F(1.19,35.71)=1.24	0,281
			b-wave	implicit time	Genotype	F(1,30)=1.55	0,222
					Genotype x flash	F(3.19,95.56)=0.409	0,758
	Fig. 2D		OPs	amplitude	Genotype	F(1,30)=1.35	0,255
					Genotype x flash	F(2.35,70.55)=0.242	0,820
	Fig. 2F	Light-adapted	b-wave	amplitude	Genotype	F(1,30)=0.0263	0,872
				implicit time	Genotype	F(1,30.0)=0.0952	0,760
mdx^{2Cv} *vs WT*	Fig. 3B	Dark-adapted	a-wave	amplitude	Genotype	F(1,30)=0.0481	0,828
					Genotype x flash	F(1.27,38.18)=4.27	0,037
			b-wave	amplitude	Genotype	F(1,30)=6.26	0,018
					Genotype x flash	F(1.46,43.77)=2.79	0,087
			a-wave	implicit time	Genotype	F(1,30)=131	<0.001
					Genotype x flash	F(1.22,36.66)=72.4	<0.001
			b-wave	implicit time	Genotype	F(1,30)=50.6	<0.001
					Genotype x flash	F(3.63,108.98)=8.13	<0.001
	Fig. 3D		OPs	amplitude	Genotype	F(1,30)=33.6	<0.001
					Genotype x flash	F(1.92,57.56)=33.6	<0.001
	Fig. 3F	Light-adapted	b-wave	amplitude	Genotype	F(1,30)=0.529	0,473
				implicit time	Genotype	F(1,23.8)=0.0418	0,840
dmd-null vs WT	Fig. 4B	Dark-adapted	a-wave	amplitude	Genotype	F(1,22)=1.01	<0.001
					Genotype x flash	F(1.59,34.88)=43.7	<0.001
			b-wave	amplitude	Genotype	F(1,24)=111	<0.001
					Genotype x flash	F(1.67,40.16)=46.0	<0.001
			a-wave	implicit time	Genotype	F(1,24)=291	<0.001
					Genotype x flash	F(1.48,35.44)=17.9	<0.001
			b-wave	implicit time	Genotype	F(1,24)=54.0	<0.001
					Genotype x flash	F(2.93,70.38)=1.43	0,243
	Fig. 4D		OPs	amplitude	Genotype	F(1,24)=64.3	<0.001
					Genotype x flash	F(2.96,71.06)=64.0	<0.001
	Fig. 4F	Light-adapted	b-wave	amplitude	Genotype	F(1,15.1)=67.8	<0.001
				implicit time	Genotype	F(1,24)=20.3	<0.001
All models compared	Fig. 5B	Dark-adapted	a-wave	amplitude	Genotype	F(3,27.2)=13.2	<0.001
			b-wave	amplitude	Genotype	F(3,27.2)=16.4	<0.001
			a-wave	implicit time	Genotype	F(3,26.9)=168	<0.001
			b-wave	implicit time	Genotype	F(3,24.9)=41.2	<0.001
	Fig. 5D		OPs	amplitude	Genotype	F(3,28.6)=26.9	<0.001
	Fig. 5F	Light-adapted	b-wave	amplitude	Genotype	F(3,26.4)=41.5	<0.001
				implicit time	Genotype	F(3,53)=3.9	0,035

Table S1. Detailed statistics of figures 2-6

Significant differences shown in red.

The left part of the table considers all eyes as independent measures (N= number of eyes), the right part shows group comparisons after averaging the two eyes for each animal (N= number of animals)

WT raw data - N = number of eyes						
**Mouse model**	**Condition**	**Wave**	**Parameter**	**Genotype, interaction**	**F**	**p**
WT-littermate groups compared	Dark-adapted	a wave	amplitude	Genotype	F(4,120) = 2.24	*0,069*
				Genotype x flash	F(5.26,157.86) = 2.87	*0,015*
		b wave	amplitude	Genotype	F(4,127) = 2.90	*0,025*
				Genotype x flash	F (6.57,208.54) = 2.89	*0,008*
		a wave	implicit time	Genotype	F(4,124) = 2.27	*0,066*
				Genotype x flash	F(4.68,114.98) = 1.17	*0,327*
		b wave	implicit time	Genotype	F(4,127) =2.72	*0,032*
				Genotype x flash	F(13.96,443.19) = 3.17	*<0.001*
		OPs	amplitude	Genotype	F (4,127) =2.63	*0,037*
				Genotype x flash	F (8.93,283.64) = 2.22	*0,021*
	Light-adapted	b wave	amplitude	Genotype	F (4,48.0) = 8.81	*<0.001*
			implicit time	Genotype	F (4,51.7) = 17.6	*<0.001*

WT raw data - N=Number of animals - (average of 2 eyes per individual)						
**Mouse model**	**Condition**	**Wave**	**Parameter**	**Genotype, interaction**	**F**	**p**
WT-littermate groups compared	Dark-adapted	a wave	amplitude	Genotype	F(4,57)=1.35	*0,261*
				Genotype x flash	F(5.24,74.69)=1.66	*0,153*
		b wave	amplitude	Genotype	F(4,61)=1.58	*0,190*
				Genotype x flash	F(6.48,98.88)=1.65	*0,137*
		a wave	implicit time	Genotype	F(4,59)=1.15	*0,343*
				Genotype x flash	F(4.65,68.63)=1.72	*0,146*
		b wave	implicit time	Genotype	F(4,61)=1.50	*0,213*
				Genotype x flash	F(13.22,201.55)=2.08	*0,016*
		OPs	amplitude	Genotype	F(4,61)=2.00	*0,106*
				Genotype x flash	F(8.76,133.66)=1.69	*0,099*
	Light-adapted	b wave	amplitude	Genotype	F(4,61)=11.6	*<0.001*
			implicit time	Genotype	F(4,24.4)=2.71	*0,054*

Table S2. Comparisons of the WT groups

Significant differences shown in red.

The left part of the table considers all eyes as independent measures (N= number of eyes), the right part shows group comparisons after averaging the two eyes for each animal (N= number of animals)

Mouse model	Condition	Wave	Parameter	Genotype effect - F value	p
mdx vs mdx^{5Cv}	Dark-adapted	a wave	amplitude	F (1,66) = 0.717	*0,400*
		b wave	amplitude	F (1,64.5) = 50.3	*0,655*
		a wave	implicit time	F (1,38.8) = 4.48e-4	*0,983*
		b wave	implicit time	F (1,48.1) = 0.856	*0,360*
		OPs	amplitude	F (1,66) = 0.819	*0,369*
	Light-adapted	b wave	amplitude	F (1,66) = 2.90	*0,093*
			implicit time	F (1,53.8) = 0.326	*0,570*
mdx vs mdx^{2Cv}	Dark-adapted	a wave	amplitude	F (1,41.8) = 10.2	*0,003*
		b wave	amplitude	F (1,60.1) = 1.46	*0,231*
		a wave	implicit time	F (1,35.9) = 173	*<0.001*
		b wave	implicit time	F (1,64) = 125	*<0.001*
		OPs	amplitude	F (1,57.5) = 24.9	*<0.001*
	Light-adapted	b wave	amplitude	F (1,55.2) = 3.35	*0,073*
			implicit time	F (1,61.2) = 0.0540	*0,817*
mdx^{5Cv} vs mdx^{2Cv}	Dark-adapted	a wave	amplitude	F (1,45.3) = 6.84	*0,012*
		b wave	amplitude	F (1,63.3) = 2.36	*0,130*
		a wave	implicit time	F (1,63.5) = 98.8	*<0.001*
		b wave	implicit time	F (1,60.0) = 76.4	*<0.001*
		OPs	amplitude	F (1,64) = 12.4	*<0.001*
	Light-adapted	b wave	amplitude	F (1,64) = 0.0971	*0,756*
			implicit time	F (1,46.5) = 0.917	*0,659*

Table S3. Statistics comparing the Dp427-deficient *mdx* and *mdx5cv* models

No differences found when normalized data from *mdx* and *mdx5cv* were compared (top table)

Same differences were found when each model was compared to the Dp260-deficient *mdx2cv* mice (signiticant genotype effects in red)
